# 

**Published:** 1909-08-14

**Authors:** 


### BOOKS RECEIVED.

Henry Frowde and Hodder and Stoughton.

"A System of Operative Surgery." Vol. IV. Edited by F. F. Burghard.

"A System of Medicine"—Vol. VI. Edited by W. Osler, M.D., F.R.S., and T. McCrae, M.D., F.R.C.P.

"The Blood in Health and Disease." By R. J. M. Buchanan, M.D., F.R.C.P.

Kegan Paul, Trench, Trubner and Co.

"Thoughts and Pastimes." By M. E. R.

### THE GRADUATES' MEDICAL SCHOOLS.

DIARY FOR THE WEEK, AUGUST 16 to 21.

**THE POST-GRADUATE COLLEGE, West London Hospital, Hammersmith, W.**

**At 10 a.m.**
**August 16 and 19,** Surgical Registrar, **Demonstration.**
**August 20,** Medical Registrar, **Demonstration,**

**At 12 noon.**
**August 16,** Dr. Bernstein, **Pathological Demonstration.**

**At 12.15 p.m.**
**August 17,** Dr. Pritchard, **Practical Medicine.**
**August 18,** Dr. Grainger Stewart, **Practical Medicine.**

**At 3 p.m.**
**August 18,** Dr. Grainger Stewart, **Medical Cases.**
**August 19,** Dr. Davis, **Medical Cases.**

**At 4 p.m.**
**August 20,** Mr. Armour, **Surgical Cases.**

**At 5 p.m.**
**August 16,** Mr. Pardoe, **Difficult Micturition and Retention of Urine.**
**August 17,** Dr. Low, **Insects as Carriers of Disease in Tropical Climates.**

### CHARITABLE BEQUESTS AND DONATIONS.

The Chelsea Hospital for Women has received from Lady Tate £1,000 to endow a bed.—The London Fever Hospital has received £100 from Lieut.-Colonel the Hon. G. W. Windsor-Clive.—The Seaside Convalescent Hospital, Seaford, Sussex, has received £1,000 from Mr. Otto Beit.—The London Skin Hospital has received £1,000 granted by the executors of the will of the late Mrs. Hannah Bland.—The late Mr. C. G. Swan left the sum of £500 to endow a cot at the Bristol Royal Hospital for Children and Women.

Mrs. Catherine Castle, of Clifton, who died in April last, left estate of the value of £55,532. She bequeathed to various persons sums amounting to £6,800, the bequests in each case to be held in trust, with remainder to the Bristol Charity Trustees. She also left £10,000 to the same charity, the yearly income of this amount to be divided, together with the income of the contingent bequests when received, into equal parts and paid annually to indigent ladies—the unmarried orphan daughters or the widows of Bristol merchants, bankers, physicians, surgeons, barristers, or solicitors. She also left £2,000 to the Bristol Royal Infirmary to endow a bed in memory of Boddam Castle, for twenty-seven years an active member of the committee, and £200 to the Clifton Dispensary.

Miss Emma Wolfe, of Jarvis Brook, Sussex, who died last month, and left estate of the value of £71,520, bequeathed, among many charitable legacies and bequests to learned societies and institutions, £1,000 to the Royal Anthropological Institute, £500 to the General Hospital, Tunbridge Wells, and £200 to the Cremation Society. She left the residue of her estate—apparently upwards of £50,000—to King Edward's Hospital Fund, the Royal Institution, Albemarle Street, and the Royal Society, Burlington House.

The Football Association, at its recent Council meeting, made the following grants to charities: Northampton Town and County General Hospital and Royal Victoria Infirmary, Newcastle-on-Tyne, £25 each; Newcastle-on-Tyne Dispensary, £20; King's College Hospital, London Hospital, Children's Hospital, Paddington Green, St. Mary's Hospital, St. Bartholomew's Hospital, St. Thomas's Hospital, and Charing Cross Hospital, £10 each; Surgical Aid Society, £7 9s. 7d.; Poplar Hospital, Royal Free Hospital, Lock Hospital, Dental Hospital, Kettering Hospital, the Blind Institution, Wellingborough Cottage Hospital, and Northampton Orphanage for Girls, £5 each.

*The Sanitary Inspectors' Examination Board.*—An examination for certificates of qualification for appointment of sanitary inspector or inspector of nuisances under Section 108 (*d*) of the Public Health (London) Act, 1891, will be held in London on Tuesday, January 18, 1910, and the four following days. In the event of a sufficient number of candidates making application, at least a month previously, examinations may also be held at the following centres: Birmingham, in June, 1910; Bristol and Cardiff, as arranged in 1910; Liverpool, in April 1910. All necessary particulars will be forwarded on application to the Hon. Secretary, the Sanitary Inspectors' Examination Board, 1 Adelaide Buildings, London Bridge, E.C.

The rate of annual subscriptions to The Hospital, either direct from the Office or through agents, is :—

| | | |
|---|---|---|
| For the United Kingdom ...... ... ... | 15s. | a year. |
| For the Colonies and Abroad ... ... ... | 17s. | ,, |
| For the United Kingdom (with Insurance Policy) ... ... ... ... ... ... ... ... | £1 | ,, |

inclusive of postage in each case.

